# Reproducibility and Validity of a Simple Checklist-type Questionnaire for Food Intake and Dietary Behavior

**DOI:** 10.2188/jea.13.235

**Published:** 2007-11-30

**Authors:** Hiroshi Yatsuya, Atsuko Ohwaki, Koji Tamakoshi, Kenji Wakai, Koji Koide, Rei Otsuka, Tomoko Mabuchi, Chiyoe Murata, Huiming Zhang, Miyuki Ishikawa, Takaaki Kondo, Hideaki Toyoshima

**Affiliations:** 1 Department of Public Health, Nagoya University Graduate School of Medicine.; 2 Nagoya Seirei Junior College.; 3 Department of Preventive Medicine, Nagoya University Graduate School of Medicine.

**Keywords:** validity of results, reproducibility of results, food group, taste preference, food habits

## Abstract

BACKGROUND: A simple, reliable, and valid food questionnaire is needed in clinical dietary assessments, community health education, and multi-purpose epidemiologic studies to obtain a crude measure of dietary intake.

METHODS: To assess the validity and reproducibility of a simple 4-point scale food intake and behavior checklist, it was compared to two 3-day weighed dietary records. The FBC was administered to 47 students of a dietician course and their parents (n=94) over a 9-month interval to assess the reproducibility. The mean intakes of selected food groups assessed by the two dietary records completed between food intake and behavior checklists were compared to the responses to the food intake and behavior checklist to assess its validity.

RESULTS: The kappa statistics for reproducibility ranged from 0.25 for confectionaries to 0.63 for a preference for fatty foods (median, 0.39). There was a reasonable level of correlation between the dietary record and the food intake and behavior checklist in the intake of eggs, milk, and fruits (r=0.53, 0.56, and 0.50, respectively). There was a weaker but still significant correlation in the intake of vegetables, and alcohol (r=0.31and 0.45, respectively). No significant correlation was observed in the intake of meat, fish, confectionaries, and soft drinks. However, those who reported consuming mainly fish rather than meat were found to eat significantly less meat and animal fat. Similarly, those who did not prefer fatty foods consumed significantly less meat, animal fat, and polyunsaturated fatty acids.

CONCLUSIONS: This simple food checklist was useful in collecting data on egg, milk, and fruit consumption. Assessing intake frequency of vegetables, meat or fish with the FBC may be useful in screening high- or low-intake individuals.

The evidence relating diet to chronic non-communicable diseases such as cardiovascular diseases, type 2 diabetes mellitus, and cancers comes from population-based epidemiologic investigations and controlled trials^1^ as well as experimental studies.^2^ There is also an increasing understanding of the roles of various nutritional components in human health and diseases. Food frequency questionnaires (FFQ) to assess nutritional intake have been widely implemented in epidemiologic studies to explore the association between diet and diseases.^3^^-^^5^ Although the standard dietary assessment instrument is more accurate, even reduced ones contain 40 to 100 items and take 15 to 30 minutes to complete,^6^^-^^11^ which is sometimes discouraging for clients. Therefore, a simple food checklist is needed that is appropriate for use in clinical dietary assessments, community health education, or multi-purpose epidemiologic studies in combination with other areas of investigation being covered.^12^ In addition to appropriateness, the checklist must be evaluated for its reproducibility and validity.

Meanwhile, dietary patterns have been realized to be important areas of investigation because of the possibility of nutrients’ interaction in disease,^13^ or the fact that the evidence regarding dietary pattern can be directly applied to reasonable dietary recommendations. Likewise, taste preferences have also gained much attention because they have been associated with patho-physiological conditions, and are considered to be an important element in modifying behavior.^14^^,^^15^

We therefore developed a simple checklist-type questionnaire for the purpose of classifying subjects into categories by obtaining a crude measure of dietary intake, which covers the intake frequency of a relatively wide range of food groups, several dietary patterns, and taste preferences while previously developed food behavior checklists mostly focused on one or two components of the diet.^16^^,^^17^ In this study, we assessed the reproducibility and validity of this questionnaire, and evaluated its usefulness and limitations by comparing it to dietary records.

## METHODS

### Food Questionnaire

The present self-administered food intake and behavior checklist (FBC) was designed to assess dietary variables that are hypothesized to affect the occurrence of cardiovascular diseases and their risk factors such as hypertension, dyslipidemia, or type 2 diabetes mellitus. The items were selected for three components of the FBC: intake frequency of food groups, taste preferences, and dietary patterns. Previous evidence regarding these components was reviewed^18^^-^^24^ to determine whether they were commonly described in the routine clinical dietary assessment and recommendations. The present FBC is a set of independent items, and, as a whole, it was not intended for the estimation of intake of specific dietary factors or summary scores. It contained 15 items: intake frequency of eight food items and alcoholic beverages (9 items), preference for specific tastes (2 items), dietary patterns or behaviors (4 items).

Food items selected for the food frequency part of the questionnaire were meat (beef, pork, or chicken), fish, eggs, milk, vegetables (the type of vegetables was not specified and both raw and cooked vegetables were included), fruits, confectionaries, and soft drinks (fruit-flavored soda with sugar, cola, canned coffee with sugar, etc.). All items on food frequency were rated on a 4-point scale (less than once per week, 1-2 days per week, 3-5 days per week, and almost every day). For simplicity, portion sizes were not asked. The questions did not specify the preceding period during which these food items were consumed.

Items selected for the preference part of the FBC were salty foods and fatty foods. Salty taste (*koi aji*) was defined in the questionnaire as that of food heavily seasoned with salt, soy-sauce or miso (fermented soybean paste). Fatty foods were not further explained in the FBC. The question on the salty taste had four possible responses: prefer and eat such foods often, prefer but abstain from such foods, prefer non-salty taste, and cannot say. The question on the fatty foods includes three possible responses: prefer fatty taste, prefer non-fatty taste, and neither.

As for the dietary patterns and behaviors, subjects were asked whether they consumed more meat or fish; frequency of eating out and not eating breakfast (the same 4-point scale as the items described above); and habit of eating to satiety. The meat or fish question originally had four possible responses: mainly meat, mainly fish, half each, and neither. Because almost no study subjects responded “neither” in the FBC, we have omitted that category in this report. The question about the habit of eating to satiety had three possible responses: eat to satiety, abstain from eating to satiety, and neither.

### Study Design and Subjects

To evaluate the reproducibility and validity of the items included in the FBC, 141 subjects were recruited from the students of the dietician course at Nagoya Seirei Junior College and their parents. Forty-seven students recruited two family members (father and mother) each. Sixty-seven percent (n=94) of the study subjects were women. The mean age and body-mass index of the fathers were 50.3 years (range: 42-59 years) and 23.0 kg/m^2^ (range: 18.1-26.1 kg/m^2^), respectively, against 19.0 years (range: 19-20 years) and 19.7 kg/m^2^ (range: 17.2-26.9 kg/m^2^) for the students, and 46.9 years (range: 39-53 years) and 22.2 kg/ m^2^ (range: 17.3-29.1 kg/ m^2^) for the mothers.

**Figure 1.**
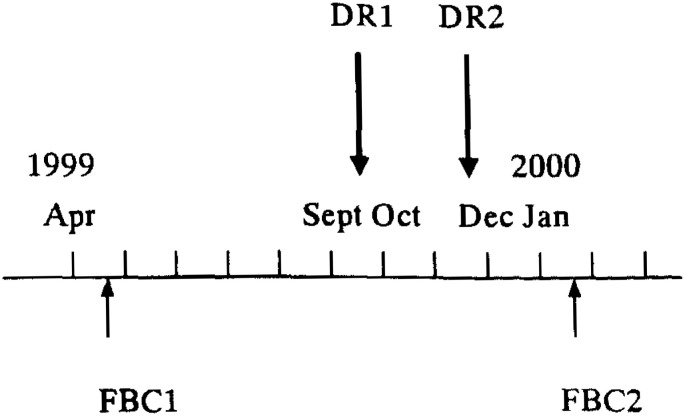
Time schedule of the dietary surveys to evaluate usefulness of the simple food intake and behavior checklist (FBC1 and FBC2) by comparing their results with those from two 3-day dietary records (DR1 and DR2)

A validation study was scheduled as illustrated in Figure 1. The study started in April 1999, when the first FBC (FBC1) was distributed to the subjects. The second FBC (FBC2) was then completed after nine months (January). Two 3-consecutive-day (which included either Saturday or Sunday, i.e., Thursday, Friday, and Saturday, or Sunday, Monday, and Tuesday) dietary records (DR1 and DR2) were completed in late September and late November, respectively. The response to FBC1 was compared to that to FBC2 to assess the reproducibility of the questionnaire, and both FBC1 and FBC2 were validated against the two 3-day DRs as the standard. However, the frequency of eating out and skipping breakfast, and the habit of eating to satiety was assessed only for the reproducibility in this report.

### Dietary Record

The weighed dietary records (DR) were completed by each subject with the assistance of the students, following a specific standardized procedure.^25^ A specially designed booklet-type printed form with instructions for completing the DR was given to each subject. The subjects were asked to describe in detail each food and the method of preparation and ingredients as well as to record all foods and beverages prepared and consumed. Dietetic scales were provided for weighing food servings. When foods or beverages could not be weighed (for example, when eating out), the subjects were instructed to describe the foods or beverages in detail, and the portion sizes were estimated from the description. These records were reviewed by one of the authors, a dietician (A.O.), to minimize variability in interpretation. The food composition table,^26^ supplemented by other sources,^27^ was used to compute nutrient and energy intake of the subjects.

### Statistical Analysis

In order to assess the reproducibility of the FBC, we calculated kappa statistics, the proportion of concordance, and the Spearman’s correlation coefficients for intake frequency between FBC1 and FBC2. The kappa statistics is defined as the agreement beyond chance divided by the amount of agreement possible beyond chance. As in most studies, kappa values of greater than 0.75 were taken to represent excellent agreement beyond chance, values between 0.40 and 0.75 fair agreement, and those less than 0.40 poor agreement.^28^^,^^29^

In the validity assessment, the selected frequency category in the FBC was converted to daily intake. The four possible responses regarding intake frequency of the FBC, specifically, less than once per week, 1-2 days per week, 3-5 days per week and almost every day, were converted to daily consumption, or 0.1, 0.21, 0.57 and 1.0 days per day, respectively. The means of two 3-day DRs were used as the individuals’ food intake.^8^ These values were either natural logarithmically or square root transformed in advance to approximately normalize their distribution. Food intake was adjusted for total energy intake, sex and age by the residual method.^30^ The Pearson’s correlation coefficients between the intake frequency of the FBC and the intake calculated from the DR were computed for each item. Correction of the observed (crude) correlation coefficient for the attenuating effect of random within-person variation (de-attenuation) was statistically performed by considering DR1 and DR2 as two independent units of observation, and by obtaining the within- and between-person variations by one-way analysis of variance (ANOVA).^31^ These correlation coefficients were also calculated by sex and age-group. Furthermore, we calculated the mean food intake from DR for 4-response categories of corresponding items on the FBC.^8^ The differences among means were tested by one-way ANOVA.

The items concerning the taste preferences and the dietary patterns were validated by calculating the mean intakes of the particular food groups and some related nutrients in each category of those questions, and the inter-categorical differences were tested by one-way ANOVA.

## RESULTS

Mean total energy intakes of the subjects were 7,334 kJ/day (1,752 kcal/day) (standard deviation, 1,193 kJ/day) for fathers, 6,222 kJ/day (1,486 kcal/day) (standard deviation, 1,037 kJ/day) for students, and 6,325 kJ/day (1,511 kcal/day) (standard deviation, 1,299 kJ/day) for mothers. Mean intake frequency per week based on the FBC1 and FBC2, and mean daily consumption of food groups (g/day) based on the two 3-day dietary records are presented for the 9 food groups in Tables 1 and 2, respectively.

**Table 1.** Mean consumption frequency (times/week) based on food intake and behavior checklists (FBC1 and FBC2) by sex and age-group (n=141).

	FBC1						FBC2					
	
	Women				Men		Women				Men	
			
	Students		Mothers		Fathers		Students		Mothers		Fathers	
	(n=47, age: 19-20 y.o.)		(n=47, age: 39-53 y.o.)		(n=47, age: 42-59 y.o.)		(n=47, age: 19-20 y.o.)		(n=47, age: 39-53 y.o.)		(n=47, age: 42-59 y.o.)	
					
	Mean	SD	Mean	SD	Mean	SD	Mean	SD	Mean	SD	Mean	SD
											
Meat	4.2	1.7	3.4	1.5	3.3	1.9	4.0	1.7	3.8	1.9	3.6	2.1
Fish	2.8	1.4	3.7	1.7	3.6	1.8	2.9	1.6	3.8	1.8	3.3	2.1
Egg	4.5	2.0	4.0	2.0	3.9	2.3	3.9	2.2	4.0	2.2	3.8	2.3
Milk	3.2	3.0	3.7	2.8	2.6	2.8	3.2	2.7	3.7	2.8	3.2	2.7
Vegetables	5.7	2.0	6.5	1.3	5.5	1.9	5.3	1.9	6.2	1.6	5.5	2.0
Fruits	3.2	2.0	4.1	2.3	2.8	2.1	3.8	2.6	4.0	2.4	3.7	2.3
Confectionaries	2.9	2.3	1.8	1.8	0.9	1.2	2.6	1.9	2.4	2.4	1.1	1.5
Soft drinks	2.6	2.3	1.0	1.7	2.6	2.8	2.5	2.0	1.3	2.2	3.0	2.7
Alcoholic beverages	0.1	0.3	1.3	2.2	4.1	3.0	0.3	0.8	1.5	2.3	3.8	3.1

SD: standard deviation

y.o.: years old

**Table 2.** Mean daily consumption of food groups (g/day) based on two three-day dietary records (DR) by sex and age-group (n=141).

	Women				Men	
	
	Students		Mothers		Fathers	
	(n=47, age: 19-20 y.o.)		(n=47, age: 39-53 y.o.)		(n=47, age: 42-59 y.o.)	
		
	Mean	SD	Mean	SD	Mean	SD
					
Meat	54	23	53	20	67	27
Fish	54	26	62	34	71	38
Egg	36	19	39	20	37	18
Milk	90	78	80	86	64	77
Vegetables	138	68	164	73	176	82
Fruits	48	44	53	53	48	52
Confectionaries	31	36	19	27	14	17
Soft drinks	335	161	396	213	353	225
Alcoholic beverages	23	48	60	159	220	281

SD: standard deviation

y.o.: years old

Reproducibility of the FBC that was administered over a 9-month interval is presented in Table 3. The kappa statistics ranged from 0.25 for confectionaries to 0.63 for a fatty food preference (median, 0.39). Though half of the items were interpreted as having fair agreement based on kappa value, nine out of 11 items (82 %) had Spearman’s correlation coefficients greater than 0.50.

**Table 3.** Reproducibility between two food intake and behavior checklists administered over a 9-month interval (n=141).

	kappa statistic	Proportion of concordance	Spearman’s correlation coefficient
Meat	0.33	0.61	0.43
Fish	0.38	0.63	0.58
Egg	0.42	0.61	0.63
Milk	0.39	0.54	0.65
Vegetables	0.22	0.62	0.37
Fruits	0.42	0.59	0.64
Confectionaries	0.25	0.51	0.53
Soft drinks	0.35	0.53	0.60
Alcohol	0.55	0.66	0.86
Salty taste preference	0.41	0.57	–
Fatty food preference	0.63	0.79	–
Meat or fish	0.43	0.65	–
Eating out	0.34	0.64	0.50
Skipping breakfast	0.42	0.82	0.62
Eating to satiety	0.34	0.60	–

Pearson’s correlation coefficients comparing daily intakes of the 9 food groups from the FBC1 and FBC2 with those from the DR are presented in Table 4. Ratios of the within-person to between-person variance components of food intake from the two 3-day DRs are also included in Table 4. Overall, the de-attenuated correlation coefficients tended to be higher in the comparison between DR and FBC2, which was collected after the administration of DR, than in that between DR and FBC1. There was a reasonable level of correlation between DR and FBC2 in the intake of egg, milk, and fruits (r=0.53, 0.56, and 0.50, respectively). There was a weaker but still significant (p<0.05) correlation in the intake of vegetables and alcoholic beverages (r=0.31 and 0.45, respectively). No significant correlation was observed in the intake of meat, fish, confectionaries, and soft drinks. Stratified analysis by sex and age-group, however, revealed that meat intakes were valid to a reasonable degree in students (r=0.51), and to a lesser degree in fathers (r=0.37, Table 5). There was also a reasonable level of correlation in the intake of alcoholic beverages in women (r=0.62 in students and r=0.59 in mothers). Intake of soft drinks in men had a weak but higher degree of validity compared to that observed in the analysis not stratified by sex and age-group (r=0.38). On the contrary, the correlation coefficient in the intake of vegetables in men was low (r=0.11) whereas those in women were comparable to the ones observed without stratification. Correlation coefficients in the intake of fish and confectionaries were lower than 0.30 in all sex- and age-groups. The number of subjects and their proportions, and mean intakes (g/day) from DR according to four response-categories of FBC1 and FBC2 are shown in Table 6. Actual intakes of vegetables by DR in those who answered in FBC2 that they consumed vegetables every day, 3-5 days per week, and 1-2 days per week were 173, 144, and 105 g/day, respectively (p<0.01, ANOVA).

**Table 4.** Pearson correlation comparing daily intakes of the 9 food groups from food intake and behavior checklist (FBC) 1 and FBC2, and the dietary records (n=141).

	*σ*_w_^2^/*σ*_b_^2^	FBC1				FBC2			
	
		Crude	Energy-, sex-, and age-adjusted			Crude	Energy-, sex-, and age-adjusted		
			
		r	r	r*	95% CI	r	r	r*	95% CI
Meat	7.23	-0.04	-0.05		NI	0.15	0.19	0.30	(-0.08 – 0.92)
Fish	2.64	0.18	0.20	0.25	( 0.03 – 0.58)	0.12	0.13	0.18	(-0.06 – 0.47)
Egg	1.90	0.22	0.22	0.34	( 0.06 – 0.56)	0.34	0.35	0.53	( 0.20 – 0.77)
Milk	0.35	0.46	0.45	0.49	( 0.30 – 0.68)	0.51	0.51	0.56	( 0.36 – 0.75)
Vegetables	0.90	0.14	0.15	0.18	( -0.02 – 0.38)	0.27	0.26	0.31	(0.10 – 0.51)
Fruits	2.22	0.32	0.36	0.49	( 0.21 – 0.84)	0.39	0.37	0.50	( 0.21 – 0.86)
Confectionaries	2.16	0.34	0.01	0.01	(-0.22 – 0.25)	0.16	0.01	0.01	(-0.22 – 0.25)
Soft drinks	1.00	-0.10	-0.08		NI	0.14	0.17	0.20	( 0.00 – 0.41)
Alcohol	0.34	0.60	0.39	0.42	( 0.24 – 0.61)	0.59	0.42	0.45	( 0.27 – 0.64)

All values were log_e_ or square root transformed.

*σ*_w_^2^/*σ*_b_^2^: Ratio of the within- to between-person variance measured from the averages of food group intake of the two 3-day dietary records.

r*: de-attenuated correlation coefficient

NI: not indicated for calculation because the coefficient was below zero.

CI: confidence interval

**Table 5.** Pearson correlation comparing daily intakes of the 9 food groups from food intake and behavior checklist (FBC) 1 and FBC2, and the dietary records by sex and age-group (n=141).

	FBC1						FBC2					
	
	Women				Men		Women				Men	
			
	Students		Mothers		Fathers		Students		Mothers		Fathers	
	(n=47, age: 19-20 y.o.)		(n=47, age: 39-53 y.o.)		(n=47, age: 42-59 y.o.)		(n=47, age: 19-20 y.o.)		(n=47, age: 39-53 y.o.)		(n=47, age: 42-59 y.o.)	
					
	r	r*	r	r*	r	r*	r	r*	r	r*	r	r*
Meat	-0.13	NI	0.03	0.06	-0.05	NI	0.29	0.51	0.05	0.10	0.21	0.37
Fish	0.19	0.29	0.27	0.41	0.14	0.22	0.04	0.07	0.19	0.28	0.15	0.24
Egg	0.18	0.28	0.24	0.37	0.25	0.38	0.35	0.54	0.34	0.52	0.33	0.50
Milk	0.66	0.71	0.31	0.33	0.44	0.47	0.49	0.53	0.49	0.53	0.57	0.62
Vegetables	0.26	0.32	-0.12	NI	0.21	0.26	0.35	0.43	0.09	0.36	0.30	0.11
Fruits	0.47	0.65	0.41	0.56	0.26	0.35	0.47	0.64	0.28	0.38	0.28	0.38
Confectionaries	0.05	0.06	0.15	0.20	0.27	0.35	0.19	0.25	0.02	0.02	-0.04	NI
Soft drinks	-0.15	NI	-0.10	NI	-0.04	NI	-0.18	NI	0.24	0.29	0.31	0.38
Alcoholic beverages	0.60	0.65	0.55	0.59	0.42	0.45	0.58	0.62	0.55	0.59	0.41	0.44

y.o.: years old

r: Pearson correlation coefficient between FBC1 or FBC2 and DR (energy-, sex-, age-adjusted intakes by the residual method)

r*: de-attenuated correlation coefficient

NI: not indicated for calculation because the coefficient was below zero.

**Table 6.** The number of subjects and their proportions, and mean intakes (g/day) from dietary records according to four response-categories of food intake and behavior checklist (FBC) 1 and FBC2.

	FBC1									FBC2								
	
	Less than once per week		1-2 days per week		3-5 days per week		almost every day		p*	Less than once per week		1-2 days per week		3-5 days per week		almost every day		p*
							
	n (%)	intake (g/day)	n (%)	intake (g/day)	n (%)	intake (g/day)	n (%)	intake (g/day)		n (%)	intake (g/day)	n (%)	intake (g/day)	n (%)	intake (g/day)	n (%)	intake (g/day)	
Meat	1 ( 0.7)	68	39 (27.7)	55	80 (56.7)	61	18 (12.8)	50	0.24	2 ( 1.4)	64	36 (25.5)	52	75 (53.2)	59	25 (17.7)	63	0.28
Fish	3 ( 2.1)	52	44 (31.2)	56	77 (54.6)	65	13 ( 9.2)	77	0.23	2 ( 1.4)	51	55 (39.0)	60	64 (45.4)	63	18 (12.8)	73	0.54
Egg	2 ( 1.4)	6	37 (26.2)	33	62 (44.0)	38	40 (28.4)	41	0.02	3 ( 2.1)	19	46 (32.6)	31	54 (38.3)	37	38 (27.0)	46	0.001
Milk	39 (27.7)	48	36 (25.5)	88	21 (14.9)	108	44 (31.2)	144	<0.001	29 (20.6)	45	38 (27.0)	66	32 (22.7)	115	42 (29.8)	146	<0.001
Vegetables	0		10 ( 7.1)	166	32 (22.7)	131	97 (68.8)	169	0.04	0		12 ( 8.5)	105	40 (28.4)	145	89 (63.1)	173	0.004
Fruits	6 ( 4.3)	58	59 (41.8)	40	48 (34.0)	64	28 (19.9)	97	<0.001	12 ( 8.5)	35	41 (29.1)	45	45 (31.9)	51	43 (30.5)	92	<0.001
Confectionaries	36 (25.5)	51	72 (51.1)	50	18 (12.8)	66	12 ( 8.5)	62	0.85	39 (27.7)	55	64 (45.4)	43	25 (17.7)	63	13 ( 9.2)	70	0.54
Soft drinks	54 (38.3)	390	46 (32.6)	365	21 (14.9)	280	20 (14.2)	362	0.22	50 (35.5)	347	39 (27.7)	351	33 (23.4)	340	19 (13.5)	456	0.19
Alcoholic beverages	81 (57.4)	23	16 (11.3)	53	11 ( 7.8)	215	26 (18.4)	334	<0.001	80 (56.7)	18	22 (15.6)	54	14 ( 9.9)	258	25 (17.7)	322	<0.001

*: p-value by one-way analysis of variance

Table 7 presents mean salt intake (g/day) according to the subjects’ preference for salty taste. The first four rows represent the salt intake measured by DR according to the four possible categories of FBC1. There was no significant difference in the intake of NaCl among the categories. Similarly, the next four rows represent the salt intake measured by DR by the four categories of FBC2. In this comparison using all subjects, the adjusted salt intake was 9.6 g/day for those who preferred and ate salty food, 9.4 g/day for those who preferred such taste but abstain, and 9.3 g/day for those who preferred a non-salty taste. Stratified analysis by sex and age-group (only women) showed that the adjusted salt intakes in both young and middle-aged women were higher in those who preferred and ate salty food (8.9 g/day and 10.2 g/day, respectively) than in those who preferred such taste but abstained (7.9 g/day and 9.2 g/day, respectively), or those who preferred a non-salty taste (8.6 g/day and 9.6 g/day, respectively). However, the differences among these means were not statistically significant. There was not any specific trend in salt intake in men.

**Table 7.**
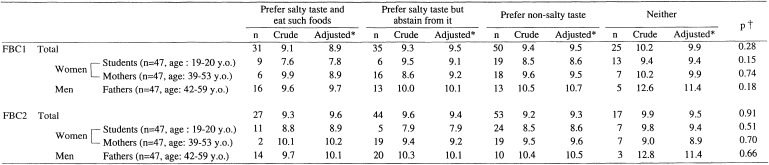
Mean sodium intake (g/day) calculated from dietary records according to the participants’ preference for salty taste.

*: Adjusted for total energy intake, sex, and age group.

FBC: food intake and behavior checklist

† : p-value by one-way analysis of variance

Mean intakes of selected food groups and nutrients related to fat metabolism (g/day) derived from DR are presented in Table 8 according to the subjects’ preference for fatty foods. Each column represents the crude or adjusted intake according to the three possible categories of either FBC1 or FBC2. There was a significant difference in meat intake (p<0.05) and a marginally significant difference in the intake of animal fat (p<0.1) and polyunsaturated fatty acid (p<0.1) between response categories of FBC2 and DR. Mean intakes of selected food groups and nutrients related to fat metabolism (g/day) according to the subjects’ meat and fish consumption are presented in Table 9. Those who answered on the FBC2 that they consumed mainly fish tended to eat less meat and less animal fat. Those who answered on the FBC1 that they consumed mainly fish tended to eat more polyunsaturated and monounsaturated fatty acids. In addition, those who answered on the FBC2 that they consumed mainly meat tended to eat less fish fat. Cholesterol tended to be consumed more by those who answered that they consumed both fish and meat.

**Table 8.** Mean intakes of selected food groups and nutrients calculated from dietary records according to the participants’ preference for fatty taste.

	FBC1							FBC2						
	
	Prefer fatty taste		Neither		Prefer non-fatty food		p ‡	Prefer fatty taste		Neither		Prefer non-fatty food		p ‡
	(n=19)		(n=41)		(n=81)			(n=23)		(n=40)		(n=78)		
					
	Crude	Adjusted †	Crude	Adjusted †	Crude	Adjusted †		Crude	Adjusted †	Crude	Adjusted †	Crude	Adjusted †	
Fish (g/day)	53.0	53.5	56.8	54.9	59.8	60.7	0.50	53.9	55.0	59.0	56.5	58.7	59.7	0.76
Meat (g/day)	65.3	65.8	59.6	58.1	56.0	56.6	0.27	65.5	66.3	66.3	64.3	52.1	52.9	0.004
Animal fat (g/day)	18.7	18.9	20.2	19.8	18.5	18.7	0.65	20.0	20.1	21.2	20.5	17.8	18.1	0.069
Fish fat (g/day)	3.8	3.8	2.9	2.8	3.7	3.7	0.16	3.3	3.3	3.4	3.3	3.6	3.6	0.84
Vegetable fat (g/day)	22.4	22.6	23.2	22.8	22.6	22.8	0.99	23.1	23.2	23.7	23.0	22.2	22.5	0.81
Cholesterol (mg/day)	242.6	242.4	259.3	256.6	252.2	253.6	0.86	263.1	265.6	260.1	255.1	246.4	248.2	0.73
Saturated fatty acids (g/day)	10.8	10.9	11.7	11.5	11.5	11.5	0.65	11.7	11.7	12.3	12.0	10.9	11.0	0.12
Polyunsaturated fatty acids (g/day)	14.3	14.4	15.6	15.5	15.3	15.4	0.47	15.4	15.4	16.6	16.3	14.6	14.7	0.067
Monounsaturated fatty acids (g/day)	10.4	10.4	11.1	10.9	10.9	10.9	0.72	10.8	10.8	11.5	11.2	10.6	10.7	0.55

† : Adjusted for total energy intake, sex, and age group.

‡ : p-value by one-way analysis of variance

FBC: food intake and behavior checklist

**Table 9.** Mean intakes of selected food groups and nutrients calculated from dietary records according to the participants’ meat and fish intake.

	FBC1								FBC2						
	
	Mainly meat		Half		Mainly fish		p ‡	Mainly meat			Half		Mainly fish		p ‡
	(n=26)		(n=78)		(n=36)			(n=37)			(n=72)		(n=30)		
					
	Crude	Adjusted †	Crude	Adjusted †	Crude	Adjusted †		Crude		Adjusted †	Crude	Adjusted †	Crude	Adjusted †	
Fish (g/day)	51.2	51.8	59.2	58.7	66.1	66.5	0.18	49.7		51.1	58.0	57.8	67.8	67.9	0.18
Meat (g/day)	57.9	58.5	61.0	60.5	51.4	51.6	0.21	59.3		60.7	60.8	60.5	51.3	51.4	0.091
Animal fat (g/day)	18.7	19.0	20.2	20.0	16.7	16.7	0.033	19.1		19.7	20.1	20.0	16.5	16.5	0.025
Fish fat (g/day)	3.1	3.2	3.5	3.5	3.9	3.9	0.55	2.7		2.7	3.8	3.8	3.8	3.8	0.066
Vegetable fat (g/day)	20.6	20.8	23.5	23.3	23.9	24.0	0.039	21.6		22.2	22.9	22.7	23.6	23.7	0.69
Cholesterol (mg/day)	231.8	231.0	268.6	268.1	245.5	246.8	0.12	234.6		235.8	271.3	270.9	234.1	235.1	0.075
Saturated fatty acids (g/day)	10.9	11.0	11.9	11.8	11.0	11.0	0.22	11.2		11.6	11.6	11.4	11.3	11.4	0.38
Polyunsaturated fatty acids (g/day)	14.0	14.1	16.0	15.9	15.1	15.1	0.034	14.6		14.9	15.7	15.6	15.0	15.0	0.23
Monounsaturated fatty acids (g/day)	9.8	9.8	11.2	11.2	11.6	11.6	0.008	10.1		10.3	11.2	11.1	11.0	11.1	0.26

† : Adjusted for total energy intake, sex, and age group.

‡ : p-value by one way analysis of variance

FBC: food intake and behavior checklist

## DISCUSSION

In this study, we found a reasonable degree of reproducibility and validity in the items measuring intake frequency for some food groups. Comparing the sex-, age-, and total energy-adjusted intake of milk, eggs and fruits with that measured by FBC yielded de-attenuated Pearson’s correlation coefficients of more than 0.50. The Spearman’s correlations representing the reproducibility of these items were more than 0.60. The question for intake of alcoholic beverages could also be considered valid and reproducible. These figures are only slightly lower than those obtained from some previous studies using more detailed FFQs, although direct comparisons are inappropriate in a strict sense because of the difference in study design. In a study using a 102-item semiquantitative FFQ for Japanese foods with an 8-point scale, de-attenuated correlation coefficients were 0.49, 0.65, and 0.54 for milk, eggs, and fruits, respectively.^6^ Our results may reflect the fact that there is a large inter-individual variation in the intake of these foods in a population of Japanese adults, which was large enough to be detected by the scale of only four points. At the same time, summary food-group questions are suggested to perform better than summing across individual foods to assess total intake, perhaps because subjects are better able to describe frequency for more generalized categories than for specific foods.^16^ Even though validity and reproducibility for these items were considered reasonable, other studies reported higher correlations.^7^^,^^10^ This may be explained by the presence of milk or egg as an ingredient in a dish. In FBC, we only assessed weekly consumption of these foods without specifying portion sizes. There might have been differences in subjects’ interpretation of the question, with some responding that they had eaten milk or eggs only when these items were consumed alone, whereas others may have considered milk and eggs included as ingredients in prepared dishes in their responses, as well as when consumed alone.

There was an insufficient level of validity in the intake of soft drinks and confectionaries. Even in a detailed FFQ, however, reported correlations for validity were 0.17 and 0.33 for beverages excluding alcohol and confectionaries, respectively.^6^ Another study conducted in Japan also reported a correlation of 0.40 for confectionaries, which was below the median correlation coefficients (0.56) observed in the same study.^7^ Low mean intake frequency of soft drinks and confectionaries in this population probably prevented us from observing meaningful inter-individual variations. In addition, subjects may have underestimated their usual intake of these foods because they are considered to be less healthy,^10^ which may have further attenuated the validity. The lack of correlation between FBC2 and DR in the intake of soft drinks in young women, i.e., the students of the dietician course, who probably have greater than average knowledge about health, may have been partly due to this possibility.

Though the reproducibility measured by kappa statistics was 0.41 (fair agreement), we did not find significant differences in salt intake among the categories of salty taste preference. Drewnowski et al. found that salty taste preference was unrelated to sodium intake.^32^ Nagata et al. found a significant, but small difference in sodium intake by preference only in women.^33^ In addition to the fact there is still uncertainty as to whether two 3-day DRs can correctly measure sodium intake,^34^ there is a possibility that some responses were affected by respondents’ knowledge of desirable answers in terms of health. It is also possible that subjects may have regarded the general Japanese term “*koi aji*” as including other tastes, such as strong or heavy tastes. Therefore, we should be careful in interpreting this result. Further studies are needed, including those using biological markers, such as measurement of urinary salt excretion or other dietary assessments.

More interestingly, those who replied that they consumed mainly fish rather than meat did eat significantly less meat and animal fat. Likewise, those who did not prefer fatty foods consumed significantly less meat, animal fat, and polyunsaturated fatty acids. These food behavior items that are not directly related to a specific food intake seemed to be surrogate measures of consumption. Similar findings were obtained by Murphy et al. using a 39-item food behavior checklist.^12^ This finding may be useful because if one food behavior can be a surrogate measure of consumption of many foods, inquiring about that specific food behavior can save time and paper. Moreover, the result of epidemiologic or clinical studies dealing with these kinds of food behavior may be directly related to dietary recommendations.

There are several limitations of the FBC. First, the maximum frequency category of the FBC was “almost every day”, which may have limited the ability of the questionnaire to discriminate between individuals with very high consumption from those with somewhat high intakes.^8^ For example, mean intake frequencies of vegetables were 0.84 and 0.86 per day in FBC1 and FBC2, respectively, and most subjects claimed to consume vegetables almost every day. The validity and reproducibility in the intake of vegetables were somewhat insufficient, and these values were lower than previous reports using fruit and vegetable modules^17^ or short food frequency questionnaires.^16^ In these two previous reports, subjects were asked to indicate the number of times vegetables were consumed per day, week, month, or year. A skewed distribution of the response seen in the present study was probably the reason for the lack of validity. However, because there was a significant difference in the mean intake of vegetables by the frequency category of the question, it is probably useful to screen low intake individuals from the population.

Similarly, correlations representing validity of the intake of meat and fish were not high. Responses to either meat or fish were skewed to middle categories, indicating that these items would be useful to discriminate, or to screen populations with extremely high or low intakes. However, for the purpose of an epidemiologic study exploring the main effect of a food on health statuses, FBC should be able to rank individuals by level of intake.^10^ Thus, information on these food items should be collected in more detail in terms of the intake frequency and serving sizes.

Second, the FBC did not specify the preceding period during which the food items were consumed. The subjects were asked to recall their usual diet since the FBCs were designed to obtain crude measures of usual diet intake. However, the lack of reference time period may have contributed to the somewhat low correlations observed because the time when the DR was carried out may not be included in the reference time period for some subjects.

Third, the FBC did not collect data on portion sizes, nor specify unit sizes of servings for each food item. It is reported that the concept of “usual” portion size varies significantly by individual,^8^ and so the lack of serving size information in several items would have limited their ability to detect between-person differences.

Other methodological issues should also be kept in mind when interpreting the present findings. First, only six days of dietary record may not accurately reflect a person’s usual diet. Therefore, we have corrected the correlation coefficient between FBC and DR by adjusting for the random within-person variation. This theoretically provides a value similar to that obtained with a large number of replicates.^31^

The study subjects were a dietician course students and their family members, and may have been more accurately understood, recalled or reported their diet than general population. This may have resulted in overestimation of the validity coefficients. It is often practically difficult to obtain DRs in a random sample of the general population, because accurately completing DR is a task requiring some knowledge.

There may have been a learning effect of keeping DR in FBC2. As shown in Table 4, the correlations between FBC2 and the DR were stronger than those between FBC1 and DR. It is possible to consider that this may be due to increased consciousness of one’s own diet through the practice of recording food intake as well as the fact that FBC is based on memory of one’s past diet.

The interval between FBC1 and DR was longer than that between FBC2 and DR. It is possible that the higher correlation coefficients observed between FBC2 and DR may be due to this difference in survey intervals.

Although we performed transformation of dietary variables to increase normality in computing the Pearson’s correlation coefficients, the criticism may be raised that a nonparametric method (e.g., Spearman’s rank correlation coefficients) should have been employed instead. Therefore, we have confirmed that this alternative method yielded an almost identical result (data not shown). We have also presented the mean intakes of each food item by the 4-response categories. This additional analysis provided analogous results regarding the validity of FBC.

The intakes of several food groups and nutrients were compared and listed according to the subjects’ preference for fatty taste and meat/fish dietary pattern. Although these comparisons were planned in advance, the possibility remains of finding chance associations due to multiple statistical tests. Therefore, biological plausibility, evidence of a dose response, and consistency of the result within and across studies must be considered in interpreting the present findings.^35^

Finally, because mean energy intakes as well as average intakes of vegetables, fruit or meat by DR are lower compared to those of same sex- and age-group from a dataset with a representative national sample,^36^ underreporting in DR may have occurred. Although the absolute intake is not of major interest in the present study, i.e., relating FBC to DR, caution is needed in interpreting the results. This is because if food groups presented for the DR did not capture important contributors to total energy intake, important associations between DR and FBC may have been missed.

In summary, the present results are of value because they help to clarify the abilities and limitations of a simple FBC. In application, the collection of data on egg, milk, fruit or alcohol intake by a simple FBC is justified. The intake of vegetables, meat and fish should be assessed with a scale having more points and questions on portion sizes. However, the FBC may still be useful in screening high or low intake individuals for these items. Asking preferences for fatty taste or assessment of food intake patterns, such as for meat or fish, may also be useful.
